# Association Between Sarcoidosis and Risk of Venous Thromboembolism: A Retrospective Chart Review

**DOI:** 10.7759/cureus.25520

**Published:** 2022-05-31

**Authors:** Ali Kassem, Loai Dahabra, Ahmad Abou Yassine, Marc Assaad, Marwah Muhammad, Dany El-Sayegh

**Affiliations:** 1 Internal Medicine, Northwell Health, Staten Island, USA; 2 Internal Medicine, Staten Island University Hospital, Staten Island, USA; 3 Internal Medicine, Jinnah Hospital, Lahore, PAK; 4 Pulmonary Critical Care, Staten Island University Hospital, Staten Island, USA

**Keywords:** inflammation, deep vein thrombosis (dvt), pulmonary emboli, hypercoagulable state, venous thromboembolism (vte), sarcoidosis

## Abstract

Introduction

Sarcoidosis is a multisystemic disorder of an unclear etiology. It has been postulated that sarcoidosis is a chronic autoimmune inflammation, which may predispose to venous thromboembolism (VTE). Recent studies showed increased VTE events in patients with sarcoidosis and other autoimmune disorders. This multicenter retrospective study aims at determining a possible correlation between VTE and sarcoidosis.

Subjects and Method

We reviewed charts from a commercial database (Explorys Inc, Cleveland, OH, USA), which is an aggregate of electronic health records from 26 major health care systems. We included patients between 30 and 69 of age. Patients with a condition known to cause a hypercoagulable state were excluded. We calculated the prevalence of VTE in patients with and without a diagnosis of sarcoidosis and compared the results. A multivariate analysis was performed to adjust for gender, race, age, tobacco use, and obesity.

Results

The overall prevalence of the VTE in patients without sarcoidosis was 1.4% compared to 4.9% in patients with sarcoidosis. Patients with sarcoidosis were more likely to develop VTE (OR: 2.96; 95% CI: 2.84-3.08; p < 0.001). Predictors of VTE in patients with sarcoidosis were gender, age, race, and obesity.

Conclusion

Our study indicates that sarcoidosis poses a risk of developing VTE. Further prospective studies are needed to shed light on this association and explain the prothrombotic phenotype of sarcoidosis.

## Introduction

Sarcoidosis is a systemic, multi-organ disease of an idiopathic cause. The clinical manifestations of sarcoidosis may vary from a self-limited disease to a disease with multi-organs involvement associated with significant mortality. Pulmonary manifestation is the most common presenting picture as it involves pulmonary parenchymal and mediastinal lymph nodes in 90% of affected patients [1,2]. It has been postulated that sarcoidosis is a chronic inflammatory disorder with immune dysregulation, as evidenced by the presence of non-caseating granuloma. Sarcoidosis can be a self-resolving or chronic illness, with episodic recurrences. Patients frequently report respiratory symptoms due to the fact that the lungs and thoracic lymph nodes are the most frequently affected organs. Less commonly, patients may present with symptoms affecting the integumentary system, eyes, or other organs given the multisystem nature of the disease [3].

This immune dysregulation may predispose to venous thromboembolism (VTE). VTE is a wide spectrum disease and encompasses deep venous thrombosis (DVT) and pulmonary embolism (PE) [4]. VTE manifests as a DVT in 66% of the cases and as a PE in 33% of the cases with or without DVT. VTE is classified as the third most common cause of vascular-related mortality in the world [4]. VTE has been increasingly reported in patients with chronic immune inflammatory disorders such as inflammatory bowel disease, systemic lupus erythematosus, and Bechet’s disease to name a few [5-7].

Recent studies have reported increased VTE events in patients with sarcoidosis [8]. While the exact mechanism of thrombosis in sarcoidosis is still not very well explained, we aimed to evaluate this possible association. In this study, we aim to determine the existence of a possible correlation between VTE and sarcoidosis and identify possible predisposing factors.

The abstract of this paper was previously posted to the American College of Chest Physicians preprint server on October 01, 2021, and published in their journal.

## Materials and methods

Our data were collected from the electronic medical record-based commercial database EXPLORYS (IMB-WATSON, Cleveland, OH, USA). This database consists of deidentified records collected from more than 26 health care systems. The database is considered Health Insurance Portability and Accountability Act compliant. Therefore, no Institutional Review Board approval was required. The data are gathered, standardized, and stored in a cloud-based system. EXPLORYS uses Systematized Nomenclature of Medicine-Clinical Terms (SNOMED-CT) for diagnosis instead of the commonly used International Classification of Diseases codes. By using the SNOMED-CT diagnosis, data can be extracted from EXPLORYS in different groups. These data can be further subdivided into smaller groups by using another diagnosis or by applying various filters such as different demographic characteristics.

A cohort of all patients with no SNOMED-CT diagnosis of sarcoidosis was set as the control group. A second cohort that included all patients with SNOMED-CT diagnosis of sarcoidosis was considered as the studied group. The prevalence of VTE was then calculated in both cohorts. Subsequently, using this database, a retrospective cohort analysis was performed including all active records from patients aged 30 to 69 years with a SNOMED-CT diagnosis of VTE between the period extending from December 2017 until December 2020.

Basic demographic information including age, sex, and race, along with tobacco use and obesity with body mass index (BMI) > 30, were identified. We excluded patients with known prothrombotic state that includes factor V Leiden, protein C/S, and anti-thrombin III deficiencies, prothrombin mutation, nephrotic syndrome, and a previous history of VTE, patients on oral contraception, and patients with a history of or active malignancy. Univariate and multivariant analyses were performed using Statistical Package for Social Sciences (SPSS), Version 25.0 (IBM Corp, Armonk, NY, USA), to adjust for the aforementioned factors. For all analyses, a p-value of less than 0.05 was considered statistically significant.

## Results

Our study population included 9,864,980 individuals, among which 33,830 patients had sarcoidosis. The overall prevalence of VTE in patients with sarcoidosis was 4.9% versus 1.4% in the general patient population (OR: 2.96; 95% CI: 2.84-3.08; p < 0.0001) (Tables 1, 2). The baseline characteristics and demographics of the study population in the sarcoidosis and general population showed that patients with sarcoidosis were more likely to be females (69%) and obese. The prevalence of obesity in sarcoidosis patients was 27% versus 12% in the non-sarcoidosis population. African Americans constituted 34% of the sarcoidosis patients and 17% of the non-sarcoidosis patients.

**Table 1:** Baseline characteristic of study population VTE, venous thromboembolism

		Sarcoidosis, N = 33,830	No sarcoidosis, N = 9,831,150
VTE		1660 (4.9%)	143,500 (1.4%)
Age group (years)	30-54	7,130	5,757,000
	55-69	26,700	4,074,150
Gender	Male	10,780 (31%)	4,407,600 (44.8%)
	Female	23,050 (69%)	5,423,550 (55.2%)
Race	Caucasians	21,860 (65%)	7,908,880 (80%)
	African Americans	11,680 (%34)	1,639,970 (17%)
	Others	290 (%1)	282,400 (3%)
Obesity BMI > 30		9,000 (27%)	1,165,080 (12%)
Active tobacco smoking		9,370 (28%)	2,989,740 (30%)

**Table 2:** Multivariate analysis with venous thromboembolism being the outcome

	OR	95% CI		p-Value
		Lower	Upper	
Sarcoidosis	2.96	2.84	3.08	0.0001
Caucasian vs African American	1.93	1.91	1.95	0.0001
Obesity	2.98	2.95	3.01	0.0001
Male vs female	1.004	0.99	1.01	0.268
Age: >55 vs <55	3.95	3.92	3.98	0.0001
Active tobacco smoking	1.87	1.85	1.89	0.0001

Both patient populations, with and without sarcoidosis, shared approximately similar percentages of tobacco smoking (28% in the sarcoidosis and 30% in the non-sarcoidosis population). Further demographics are presented in Table 1. In our study, in the multivariate analysis, it was found that patients with sarcoidosis were more likely to develop VTE compared to the general population (OR: 2.96; 95% CI: 2.84-3.08; p < 0.0001). Other than sarcoidosis, predictors of VTE in our patient population included age older than 55 years, obesity, Caucasian race, and active tobacco smoking. Age older than 55 years had the highest correlation with and incidence of VTE (OR: 3.95; 95% CI: 3.92-3.98; p < 0.0001) (Table 2). In the multivariate analysis, patients who were obese, older than 55 years, and tobacco users had a higher risk of developing VTE, with obesity being the strongest predictor (OR: 2.01; 95% CI: 1.85-2.19) (Table 3).

**Table 3:** Predictors of venous thromboembolism in patients with sarcoidosis (multivariate analysis)

	OR	95% CI		p-Value
		Lower	Upper	
Caucasian vs African American	1.00	0.93	1.09	0.836
Obesity	2.01	1.85	2.19	0.0001
Male vs female	1.04	0.96	1.13	0.268
Age: >55 vs <55	1.77	1.64	1.92	0.0001
Active tobacco smoking	1.34	1.22	1.48	0.0001

## Discussion

Coagulation and innate immunity have a common evolutionary origin; therefore, it is not surprising for autoimmune diseases to be associated with VTE, and several mechanisms can be disrupted as a result of immune dysregulation [9,10]. The exact pathophysiology behind the increased risk of VTE in sarcoidosis is still uncertain. Interestingly, it was observed in many studies that the development of thrombosis happens in the anatomic vicinity of the ongoing inflammation, for instance, mural thrombus in myocardial sarcoidosis [11], cerebral vein thrombosis in neurosarcoidosis [12-14], thoracic vein thrombosis in mediastinal sarcoidosis [15-17], along with many other organs and locations. This highly proposes the possible presence of a local hypercoagulable state that promotes the formation of thrombosis, likely secondary to the local inflammation imposed by the disease process. Another possible etiology that can explain thrombosis is cytokine regulation. The granulomas that are considered to be the hallmark of the disease contain several inflammatory cells, including macrophages that secrete cytokines that themselves maintain the granulomatous formation [18,19]. Cermak et al. proved that high levels of tissue factor (TF) can be synthesized with a subsequent high procoagulant potential when peripheral blood monocytes are incubated with highly purified (> 90%) human CRP (C-reactive protein) for 6 hours [20]. An identical phenomenon is thought to be possibly accountable for the increased procoagulant capacity of the granulomatous inflammation in this disease. Furthermore, it has been observed that patients with sarcoidosis, as compared to control individuals, had an intensified activity of TF and plasma factor VII in their bronchoalveolar lavage (BAL) fluid sample [21], which can also explain the higher hypercoagulable state, especially within the lungs. Also, other studies showed that the secretions and fluids collected by BAL in individuals with pulmonary sarcoidosis had a significantly increased procoagulant activity [22-24]. Further experiments confirmed that the amounts of fibrin split products (i.e. D-dimer) in the serum and within sampled BAL are significantly elevated in patients with pulmonary sarcoidosis [25,26]. Other risk factors include chronic steroid resulting in obesity and disabling sarcoidosis in patients with pulmonary fibrosis and pulmonary hypertension. There might be also a coexistence of thrombophilia to play a role in pathogenesis, as such one study reported that 38% of sarcoidosis patients tested positive for antiphospholipid antibodies [27]. Our study proves the presence of a significant correlation between sarcoidosis and VTE. Sarcoidosis possibly induces VTE through different inflammatory and biochemical mechanisms. Future studies, perhaps at the molecular and biochemical levels, can help establish the precise pathophysiology and subsequently suggest preventive measures and possibly different therapeutic approaches.

To our knowledge, our study is one of the largest database studies that show a significant association between sarcoidosis and VTE. The results show that 1,660 (4.9%) patients with sarcoidosis had VTE, which is significantly higher than VTE incidence in the general population. The results reveal that patients with sarcoidosis were more likely to develop VTE than patients without sarcoidosis (OR: 2.96; 95% CI: 2.84-3.08; p < 0.0001) (Table 2). After adjusting for multiple factors, these findings are consistent with other studies that also confirmed a higher incidence of VTE in patients with sarcoidosis when compared to the general population [28,29]. For instance, a study from the United Kingdom showed higher risk of PE in patients with underlying sarcoidosis, with a reported risk ratio of 2.0 [30]. Moreover, Swigris et al. showed that 2.5% of patients with sarcoidosis who died between the years 1988 and 2007 in the United States had PE listed as the cause of death [31]. The study conducted by Ungprasert et al. showed that the risk of incidence of VTE after adjusting for multiple factors was still significantly higher among patients with sarcoidosis, with a hazardous ratio (HR) of 3.04 (95% CI: 1.47-6.29) [28]. Additionally, a significantly higher risk was seen in both subtypes of VTE, with an HR of 3.14 (95% CI: 1.32-7.48) for DVT and an HR of 4.29 (95% CI: 1.21-15.23) for PE [28].

The major strengths of the study is the large number of patients included and the use of comprehensive database that allow to capture all recognized cases of sarcoidosis and VTE coded in the studied population; also, this approach minimize the referral bias and also reveals significant results after adjusting for confounding factors, as we were able to detect an augmented overall risk of VTE within the sarcoidosis group as well as determine specific risk factors and predictors for the higher incidence of VTE in the sarcoidosis group. For example, our study shows that obese patients with sarcoidosis had an OR of 2.01 (95% CI: 1.85-2.19; p < 0.0001) (Table 3) even after adjusting for confounding factors. To this day, no other study showed this specific correlation. However, in the literature, it was reported that patients with sarcoidosis have a higher BMI compared to that in the general population, which is associated with poorer outcome [32]. Obesity is a known risk factor for VTE, and its presence in the background of chronic inflammation and immune dysregulation might poses higher risk for VTE. This also might be explained by the use of steroids as a first-line therapy and decreased physical activity secondary to advanced stage of the disease. Moreover, according to our study, age was another predictor factor. In fact, our data show that patients older than 55 years with underlying sarcoidosis had an OR of 1.77 (95% CI: 1.64-1.92; p < 0.0001) (Table 3), making it the second strongest risk factor for VTE in sarcoidosis. Additionally, smoking was also shown to be an additional risk factor for VTE in the sarcoidosis population, with an OR of 1.34 (95% CI: 1.22-1.48; p < 0.0001) (Table 3). It is noteworthy to state that gender and race did not impose a higher risk of VTE in sarcoidosis patients, which was similarly described by Swigris et al. [31].

Limitations to our study include the fact that the study is a retrospective data analysis with the lack of differentiating patients based on stages of pulmonary sarcoidosis. Another limitation is the inability to exclude drug-induced VTE, for instance, steroids, monoclonal antibodies, and immunotherapy, which are used in more refractory cases and might also be contributing factors.

## Conclusions

Our study showed the presence of a significantly higher risk of VTE associated with sarcoidosis. The importance of detecting the association between sarcoidosis and VTE lies in the ability of raising awareness of this association especially among medical professionals, as early detection and subsequent immediate treatment of this possible comorbidity can positively affect morbidity and mortality and help provide optimal medical care for these patients. Further prospective studies should be conducted to take all these factors into consideration. Meanwhile, higher awareness should be applied to obese patients with underlying sarcoidosis.
